# Tumor microenvironment-mediated immune tolerance in development and treatment of gastric cancer

**DOI:** 10.3389/fimmu.2022.1016817

**Published:** 2022-10-20

**Authors:** Yuanda Liu, Changfeng Li, Yaoping Lu, Chang Liu, Wei Yang

**Affiliations:** ^1^ Department of Endoscopy Center, China-Japan Union Hospital of Jilin University, Changchun, China; ^2^ Department of Immunology, College of Basic Medical Sciences, Jilin University, Changchun, China

**Keywords:** immune tolerance, tumor microenvironment, gastric cancer, immunotherapy, tumor-infiltrating immune cells

## Abstract

Tumor microenvironment is the general term for all non-cancer components and their metabolites in tumor tissue. These components include the extracellular matrix, fibroblasts, immune cells, and endothelial cells. In the early stages of tumors, the tumor microenvironment has a tumor suppressor function. As the tumor progresses, tumor immune tolerance is induced under the action of various factors, such that the tumor suppressor microenvironment is continuously transformed into a tumor-promoting microenvironment, which promotes tumor immune escape. Eventually, tumor cells manifest the characteristics of malignant proliferation, invasion, metastasis, and drug resistance. In recent years, stress effects of the extracellular matrix, metabolic and phenotypic changes of innate immune cells (such as neutrophils, mast cells), and adaptive immune cells in the tumor microenvironment have been revealed to mediate the emerging mechanisms of immune tolerance, providing us with a large number of emerging therapeutic targets to relieve tumor immune tolerance. Gastric cancer is one of the most common digestive tract malignancies worldwide, whose mortality rate remains high. According to latest guidelines, the first-line chemotherapy of advanced gastric cancer is the traditional platinum and fluorouracil therapy, while immunotherapy for gastric cancer is extremely limited, including only Human epidermal growth factor receptor 2 (HER-2) and programmed death ligand 1 (PD-L1) targeted drugs, whose benefits are limited. Clinical experiments confirmed that cytotoxic T-lymphocyte-associated protein 4 (CTLA-4), vascular endothelial growth factor receptor (VEGFR) and other targeted drugs alone or in combination with other drugs have limited efficacy in patients with advanced gastric cancer, far less than in lung cancer, colon cancer, and other tumors. The failure of immunotherapy is mainly related to the induction of immune tolerance in the tumor microenvironment of gastric cancer. Therefore, solving the immune tolerance of tumors is key to the success of gastric cancer immunotherapy. In this study, we summarize the latest mechanisms of various components of the tumor microenvironment in gastric cancer for inducing immune tolerance and promoting the formation of the malignant phenotype of gastric cancer, as well as the research progress of targeting the tumor microenvironment to overcome immune tolerance in the treatment of gastric cancer.

## Introduction

Gastric cancer (GC) is one of the most common digestive tract malignancies worldwide, ranking fifth in morbidity and fourth in mortality (1). With the development of early diagnosis technology, although the incidence of GC exhibits a certain downward trend, the fatality rate of patients at an advanced stage that is inoperable is very high, and there is no effective treatment plan to date. In recent years, the rise of tumor immunotherapy has fueled the last hope for patients with advanced GC. Currently, the only targeted immunotherapy regimens for GC are Human epidermal growth factor receptor 2 (HER-2) monoclonal antibody, programmed death 1 (PD-1) monoclonal antibody and programmed death ligand 1 (PD-L1) monoclonal antibody. However, in GC patients, only 15–30% of patients are HER-2 positive, and the benefits of the treatment are limited (2). Although the efficacy of the PD-L1 monoclonal antibody is superior to first-line chemotherapy, the overall median survival of patients is only extended by two months. This may be related to the existence of immune tolerance in some patients (3). Chimeric Antigen Receptor T-Cell (CAR-T) therapy for GC is currently limited to clinical trials and a few case reports. An effective anti-tumor immune response includes effective presentation of antigens by dendritic cell (DC) cells, the activation and proliferation of specific T cells, and the maintenance of a lasting immune response. Inhibition of any of these points will lead to immune tolerance of the tumor (4). Therefore, in-depth exploration of the mechanism of immune tolerance in GC will help develop more effective treatment options.

Tumor microenvironment (TME) is the general term for all non-cancer components and their metabolites and secretions in tumor, which includes a large number of immune infiltrating cells, such as immune infiltrating lymphocytes (TILs). These immune cells constitute the immune microenvironment of the tumor. Current studies confirmed that TME has an important impact on malignant phenotypes such as tumor growth, invasion, metastasis, drug resistance, and immune escape. Stomach has a strong acidic environment and a unique endocrine system, which also makes the tumor microenvironment of gastric cancer different. Tumor immune microenvironment has both tumor-promoting and tumor-suppressing effects. In the stage of tumorigenesis, TME has a tumor-suppressing effect. However, as the tumor progresses, components of the tumor-suppressing microenvironment are continuously inhibited, and the tumor-promoting microenvironment is constantly being suppressed, leading to immune tolerance and tumor progression. In the process of tumor progression, on the one hand, tumors inhibit the function, number, and distribution of cytotoxic immune cells in the tumor microenvironment by competing for metabolites, secreting extracellular vesicles and cytokines, reducing the expression of self-antigens, resulting in immune tolerance. A large number of cancer-promoting immune cells continue to dominate tumors, which accelerates tumor progression and further inhibits the function of cytotoxic immune cells. Targeting the tumor microenvironment to inhibit the positive feedback loop of tumor immune tolerance is expected to contribute to a better treatment of tumors. In this article, we summarize the latest mechanisms of cellular components in the tumor microenvironment of GC for inducing immune tolerance, promoting the formation of the malignant phenotype of GC, and targeting the components of the tumor microenvironment to reduce immune tolerance in the research progress on the treatment of GC.

## The constitution of GC TME

The tumor microenvironment of GC is composed of extracellular matrix (ECM), fibroblasts, endothelial cells, mesenchymal stem cells, macrophages, lymphocytes, neutrophils and other cell components. The metabolites and cytokines secreted by these cell components (including GC cells) are also important components of TME. These components in GC TME play their own roles in inducing the immune tolerance to promote the GC progress.

### Tumor-associated macrophages

**Figure 1:**
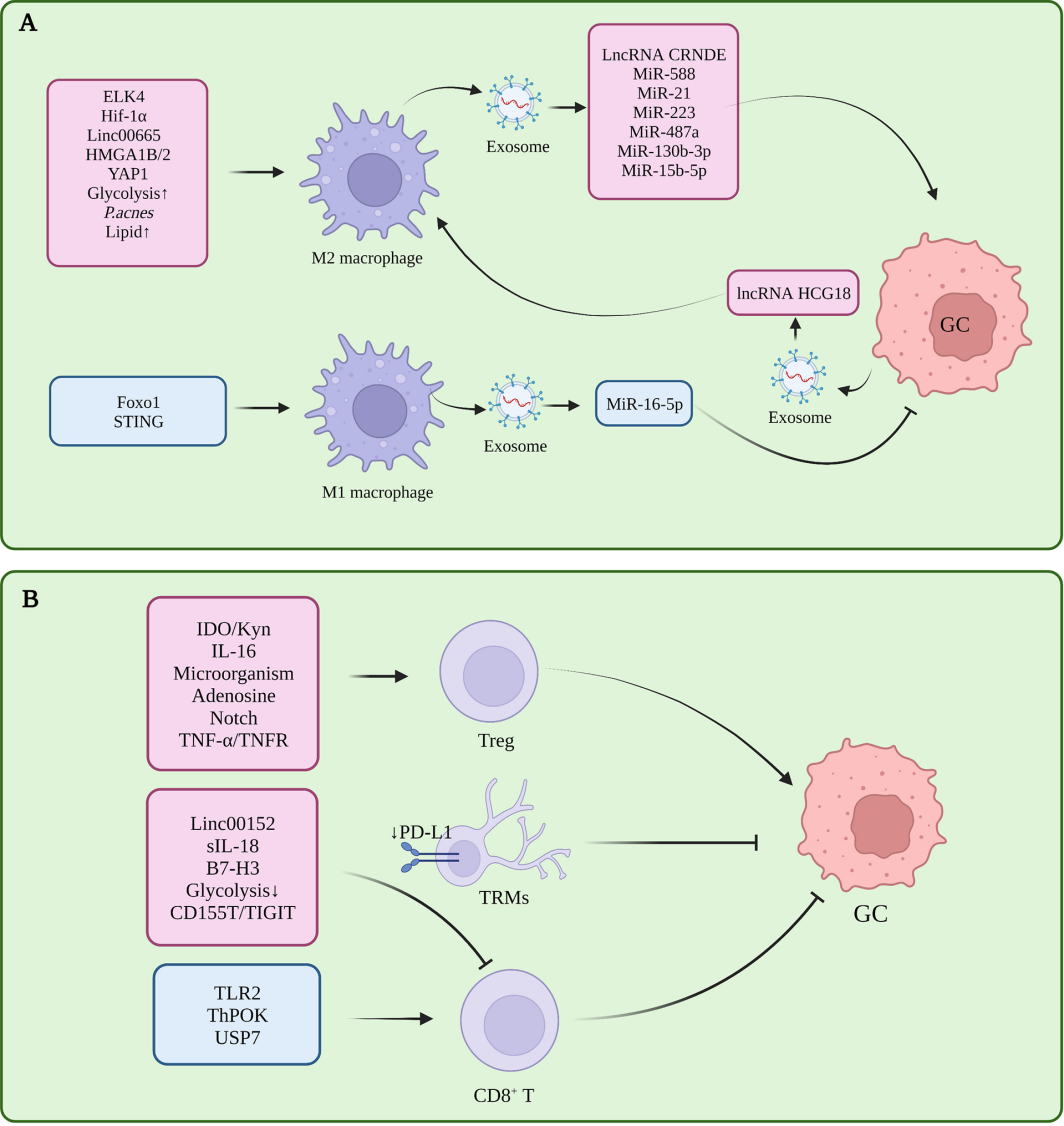
**(A)** Tumor-associated macrophages in gastric cancer immune tolerance. **(B)** Tumor-infiltrating T cells in gastric cancer immune tolerance.

Macrophages infiltrating the tumor microenvironment are called tumor-associated macrophages (TAMs), which have two polarization-activated states, namely, classical M1 polarization with tumor suppressor function and alternatively-activated M2 polarization with tumor-promoting function (5). In GC, M2 polarization of TAM is induced in the tumor microenvironment, and inhibition of M1 polarization is one of the important factors in the formation of immune tolerance (**Figure 1A**).

Several studies had proved that several molecules participated in M2 polarization in TME, which is closely related to the progress of GC. Pentraxin-3 (PTX3) can inhibit the stemness of GC cells and M2 polarization of macrophages, and prevent the formation of papillary metastases in GC (the early stage of ascites metastasis) (6). ETS-like transcription factor 4 (ELK4) promotes M2 polarization of macrophages by activating lysine-specific demethylase 5A (KDM5A), which inhibits the expression of Praja2 (PJA2) by removing H3K4me3 of the PJA2 promoter, thereby promoting M2 polarization of macrophages (7). Cisplatin induced activation of hypoxia inducible factor 1 alpha subunit (HIF1α) signaling directly drives the transcription of tumor-derived leukemia inhibitory factor, activates the STAT3 signaling pathway, and stimulates M2 polarization of macrophages, thereby promoting the resistance of gastric tumors to chemotherapeutic drugs (8). POU class 1 homeobox 1 (POU1F1) upregulated by High mobility group A 1B/2 (HMGA1B/2) promotes GC metastasis by regulating macrophage M2 polarization in a Chemokine 12 (CXCL12)/CXC motif chemokine receptor type 4 (CXCR4) dependent manner (9). Propionibacterium acnes (*P. acnes*) promotes gastric cancer progression by promoting M2 polarization of macrophages through Toll-like Receptor 4 (TLR4)/phosphoinositide 3-kinase (PI3K)/protein kinase B (AKT) signaling (10). Calmodulin 2 (CALM2) in GC promotes M2 polarization of macrophages through the Adenylate kinase 2 (AK2)/Signal transducer and activator of transcription 3 (STAT3)/HIF-1/vascular endothelial growth factor A (VEGFA) axis, thereby promoting GC metastasis and angiogenesis (11). Therefore, these molecules or signal pathway related to the M2 polarization might be the potential targets for treating GC effectively.

In addition, CD68^+^ CD163^-^ M1 macrophages are required for PD-1/PD-L1 monoclonal antibody using in GC treatment (12). Interestingly, the knockdown of STING in THP1 cell line or activation of STING *via* 2’3’-c-GAMP were shown to promote M1 polarization of macrophages and exert an anti-tumor effect, suggesting that the STING pathway has complex and meaningful regulatory roles in macrophages (13).In gastric cancer, macrophages can induce the transformation of mesenchymal stem cells (MSC) cells into fibroblasts, and then participate in the formation of immune tolerance (14).

In fact, macrophages are an emerging tumor therapeutic target, and current therapeutic modalities for TAM include the inhibition of macrophage recruitment in tumors, depletion of macrophages, induction of macrophage reprogramming to the M1 phenotype, and enhanced phagocytosis of macrophages (15). We look forward to future studies that can demonstrate the critical role of TAMs in GC. Currently, CAR-macrophages have entered the phase I clinical trial stage as the latest CAR cells, but their application in GC remains lacking (16).

### T cells

T cells are highly heterogeneous. In TME, CD8^+^ T cells assume the role of killing tumor cells, while Treg is the most representative CD4^+^ immunosuppressive cell. In addition to memory T cells, γδ T cells, Nature killing (NK) T cells, and Th cells have been shown to play an important role in tumor progression and immune tolerance in gastric cancer (**Figure 1B**).

The decrease in the number and dysfunction of CD8^+^T cells is one of the reasons for gastric cancer immune tolerance. In GC tissues with high expression levels of B7-H3 (CD276), the density of CD8^+^ T cells within the tumor was reduced, suggesting that B7-H3 may be involved in the mechanism of tumor evasion of immune responses (17). Toll-like receptor 2 (TLR2) was down-regulated in CD8^+^ T cells of gastric cancer patients, and TLR2 activation could increase the expression of perforin and granzyme B in CD8^+^ T cells and enhance CD8^+^ T cells cytotoxicity (18). The chromatin status of tumor-specific T cells is correlated with their dysfunction (19), and GC patients with high open circulating CD8^+^ T cell chromatin respond better to pembrolizumab (20). CD103^+^ CD4^+^ T cells exhibit an immunosuppressive phenotype and high retention capacity in GC tumor tissues, leading to CD8 ^+^ T cell dysfunction, and granzyme B (GZMB), interferon-γ (IFN-γ), tumor necrosis factor α (TNF-α), and perforin (PRF-1) reduction (21).

In an *in vitro* 3D culture model, Treg cells were enriched in early intestinal-type GC and could promote the growth of spheroids by inducing interleukin-2Rα (IL-2Rα) expression and activation of mitogen-activated protein kinases (MAPK) signaling pathway in tumor cells (22). The infiltration level of tumor necrosis factor receptor 2 (TNFR2)^+^ Tregs increases with the progression of GC. This is a prognostic marker and independent risk factor for GC, and activation of the TNF-α/TNFR2 pathway promotes the immunosuppressive phenotype and function of Tregs (23). Gastric mucosal microbial analysis found that Comamonas and Gaiella were negatively correlated with the number of pDCs and Tregs in GC, and Stenotrophomonas and Selenomonas were positively correlated with the number of pDCs and Tregs in GC, revealing the impact of microorganisms on tumor immunity (24). DAPT, a γ-secretase inhibitor that inactivates Notch signaling, can reduce the immunosuppressive capacity of CD4^+^CD25^+^CD127 ^dim/-^ Tregs after DAPT treatment in GC (25). CD4^+^ T cells in GC can promote the up-regulation of PD-L1 in mesenchymal stem cells through p-STAT3, thereby stimulating the proliferation of GC cells. This further stimulates the proliferation of GC cells. However, this study did not specifically explore the subset of CD4^+^T functions, and the role of Treg remains to be elucidated (26). Therefore, Treg cells infiltrated in GC tissue play an important role in the progression of the disease by inducing immune tolerance. By targeting the inhibition of Treg production or function, this may relieve the immune tolerance state of GC patients, leading to a more effective delay or treatment of the disease.

The zinc finger and BTB domain containing 7B (Zbtb7b, Alias ThPOK) as transcription factors can upregulate sperm tail PG-rich repeat containing 1 (STPG1) and downregulate Tumor necrosis factor receptor superfamily member 12A (TNFRSF12A) at the transcriptional level, inhibiting the proliferation of gastric cancer cells and promoting the proliferation of T cells (27, 28). The CXXC zinc finger protein 4 (CXXC4) can activate T cells by inhibiting the ETS-like transcription factor 1 (ELK1)/MIR100HG pathway, increase the IFN-γ secreted by CD3^+^ T cells, and relieve the immune tolerance of GC cells (29). Dexamethasone can inhibit immune evasion by inducing T cell glucocorticoid receptor (GR)/STAT3 pathway-mediated downregulation of PD-L1 and Indoleamine 2,3-dioxygenase 1 (IDO1) (30). In GC, ubiquitin-specific processing protease 7 (USP7) directly interacts with PD-L1 to stabilize it. USP7 inhibitors likewise inhibit tumor proliferation and promote PD-1/PD-L1 expression and immune response (31).

T cells are the executors of tumor immunity, as they directly exercise the tumor-killing function. In the context of inducing immune tolerance in the tumor microenvironment, CD8^+^ T cells appear dysfunction and exhausted, and immune checkpoint inhibitors against CD8^+^ T cells appear as an inefficient method. Therefore, reversing the immune tolerance microenvironment in TME and restoring the number, infiltration range, and function of CD8^+^T cells are the most popular solutions to reduce immune tolerance.

### Neutrophils

**Figure 2:**
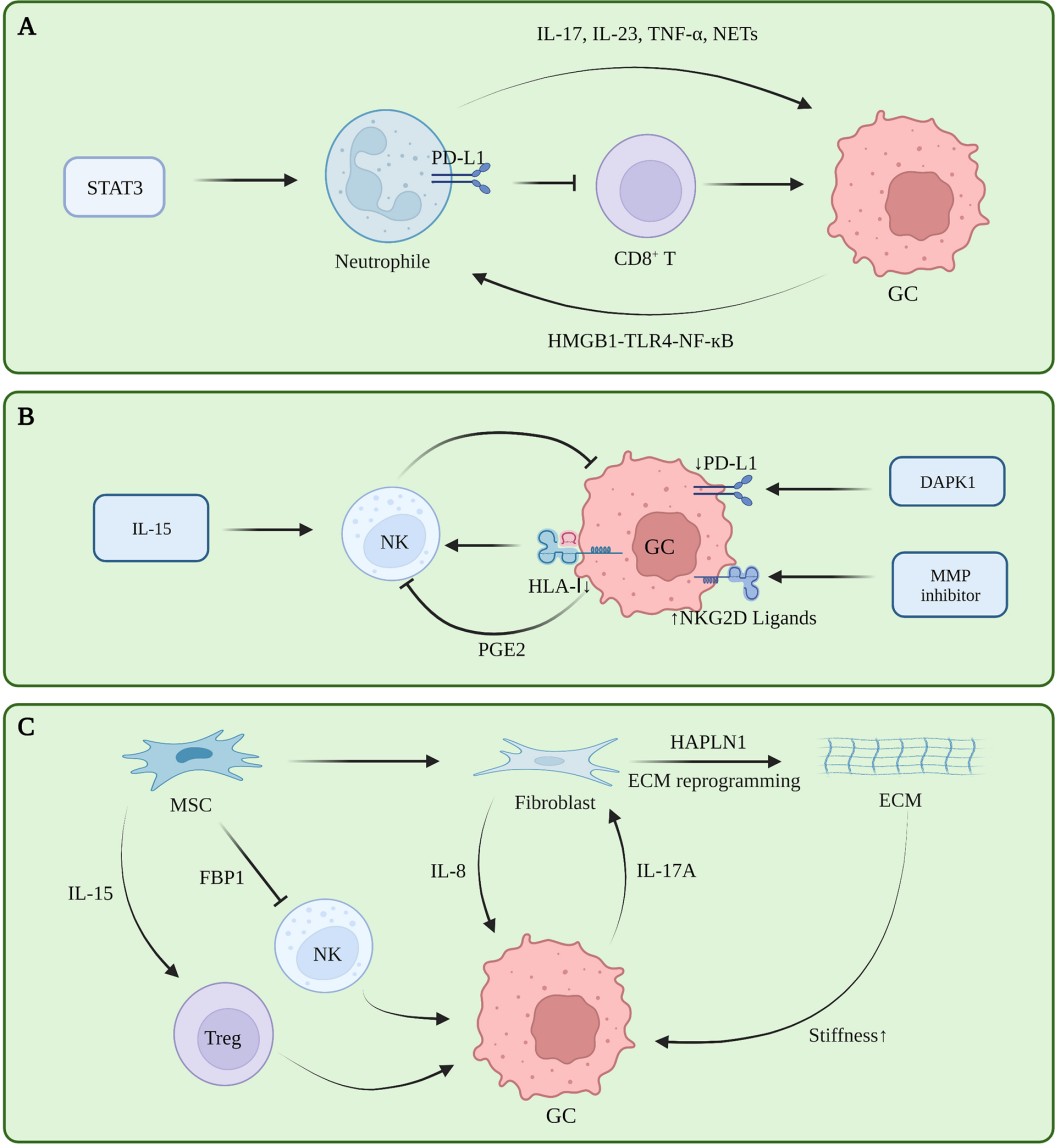
**(A)** Tumor-associated neutrophils in gastric cancer immune tolerance. **(B)** NK cells in gastric cancer immune tolerance. **(C)** ECM, fibroblasts and mesenchymal stem cells in gastric cancer immune tolerance.

Tumor-associated neutrophils (TAN) are functionally classified as tumor-suppressing N1 cells and tumor-promoting N2 cells. Transforming growth factor beta (TGF-β) induces N1 to N2 polarization (32). A retrospective study showed that a large number of tumor-associated neutrophils infiltrating GC tissue indicate a greater the risk of lymph node metastasis (33). TANs promote the progression of GC by promoting the polarization of IL-17A producing Th subset cells through the B7-H2-extracellular signal-regulated kinase (ERK) pathway (34). In human neutrophils and GC cells co-culturing experiments, blocking the formation of NETs regulates the expression of Bcl-2, Bax, and nuclear factor kappa B (NF-κB) in GC cells, promoting GC cell apoptosis and inhibiting their invasion (35, 36). Tumor-derived GM-CSF activates neutrophils and induces PD-L1 expression in neutrophils through the Janus kinase (JAK) signaling and activator of STAT3 signaling pathway. Activated PD-L1^+^ neutrophils effectively suppress normal T cell immunity *in vitro* and promote human GC growth and progression *in vivo* (37). The FasL (CD95L)^+^ PD-L2^+^ neutrophil subpopulation accounts for more than 20% of all neutrophils in advanced GC. This conditional neutrophil (TCN) was treated with FasL and/or PD-L2 antibodies. After treatment, the injection of CD8^+^ T cells into tumor-bearing mice constructed with SGC-7901 can significantly reduce the tumor volume and increase the infiltration of CD8^+^ T cells, indicating that this subset of neutrophils is involved in gastric cancer. This has a significant immunosuppressive effect against CD8^+^ T cells (38). *In vitro* co-culture experiments and IHC showed that TAN infiltrates PD-1^+^ T cells, inhibits T cell proliferation, up-regulates the expression of PD-L1, and promotes the formation of an immunosuppressive microenvironment (39). Current studies showed that TAN promotes tumor immune tolerance in tumors by remodeling the ECM, promoting angiogenesis, generating NETs, and interacting with other immune cells (40). Currently, there are also some therapeutic regimens targeting neutrophils to relieve immune tolerance. However, reducing the risk of infection caused by neutrophil levels is still the biggest obstacle to this regimen **(** **Figure 2A** **)**.

### NK cells

NK cells can directly kill target cells and recognize tumor cells that CD8^+^ T fails to recognize. However, NK cells exhibit dysfunctional behavior in TME (41). The infiltration level of NK cells in tumors and the level in peripheral blood are positively correlated with the prognosis of GC patients, and negatively correlated with the expression level of cyclooxygenase-2 (COX-2) (42, 43). The c-myc of NK cells in the peripheral blood of GC patients is down-regulated at the RNA and protein levels, and mitotic arrest is associated with NK dysfunction in GC patients (44). The expression level of the NK activating receptor NK Group 2 Member D (NKG2D) in GC patients is positively correlated with patient prognosis. Although NK cells in the resting state have little cytotoxicity against GC, NK cells induced by the K562-mb15-41BBL cell line *in vitro* have strong effects on GC cytotoxicity and strong antitumor activity in animal experiments (45). Decreased human leukocyte antigen class I (HLA-I) expression leads to decreased NK cell infiltration in GC and is insensitive to NK cell activity (46). Death-associated protein kinase 1 (DAPK1) downregulates the IKKβ/CSN5 axis in GC, inhibits PD-L1 expression, and activates the killing ability of NK cells (47). Matrix metalloproteinase (MMP)-2, MMP-9, and pan-MMP inhibitors can upregulate the expression of NKG2D ligands in GC, making GC cells more sensitive to NK cells (48). In *in vitro* experiments, prostaglandin 2 (PGE2) secreted by GC cells can inhibit the proliferation of NK cells and induce their apoptosis (42). IL-15 can activate the activity of NK cells and inhibit the formation of liver metastases in a mouse model of GC liver metastasis (49). iNKT cells are involved in the initial steps of anti-tumor immunity. However, the increased frequency of iNKT in peripheral blood of patients with GC does not bring good benefits to the patients. Subsequent experiments have shown that the ability of iNKT cells to degranulate and produce IFN-γ in patients with GC is impaired (50). Further follow-up studies are required to clarify the heterogeneity of NK cells in GC, and the factors that inhibit NK cell function in GC TME, to find an effective NK cell-based therapeutic regimen for GC **(** **Figure 2B** **)**.

### ECM

The ECM is a network of collagen, fibronectin, laminin, vitronectin, elastin, as well as growth factors, cytokines, and matrix metalloproteinases that support and maintain the epithelial cell structure (51). These components are mainly secreted by fibroblasts, although other cells in the microenvironment likewise have the ability to secrete these substances (52). In the stage of gastric carcinogenesis, ECM is considered to be an initiating factor of gastric carcinogenesis. Studies showed that different subtypes of GC have different ECM components, and that a lower degree of differentiation indicates a greater abundance of ECM components, higher cell metabolism, and higher degree of metabolic reprogramming (53). Proteomic analysis revealed that ECM components of tumor tissues were no different from normal tissues, whereas their levels varied greatly, which was mainly manifested as increased ECM proteins and decreased basement membrane components that were closely related to tumor angiogenesis, invasion and metastasis, i.e., closely related to the formation of malignant phenotypes (54). During the progression stage of GC, ECM deposits continuously and increases in density, directly interacting with receptors on the surface of tumor cells, reducing E-cadherin/β-catenin, and promoting the proliferation, invasion, and metastasis of GC cells (55). Further, enhanced environmental stress caused by the increased matrix density is likewise an important reason for ECM to promote tumor progression. The researchers cultured GC cells in hydrogels with different stress intensities and found that with the increase of stress, the CD44 expression of tumor cells reversibly became nonfunctional. isomers, promoting the metastasis of GC (56). In fact, the stress role of ECM in breast cancer has been confirmed, and high-strength ECM can promote the epithelial-mesenchymal transition (EMT) process of breast cancer, increase the infiltration of M2 macrophages, and inhibit the function of CD8^+^ T cells (57, 58). However, this mechanism must be further clarified in GC **(** **Figure 2C** **)**.

### Fibroblasts

Cancer associated fibroblasts (CAF) are the main cells that secrete and degrade ECM, and also secrete a large number of cytokines, chemokines, and exosomes. In gastric cancer, hyaluronan and proteoglycan link protein 1 (HAPLN1) were the most significantly upregulated genes in fibroblasts. Second harmonic generation imaging with a multi-photon microscope showed that the knockdown removal of HAPLN1 significantly reduces the density, length, width, and number of fibers in the ECM (59). A study on secretomes revealed that a Helicobacter pylori infection can lead to changes in the secretion of fibroblasts in the gastric mucosa, induce metabolic reprogramming and changes in the microenvironment of epithelial cells and tumor cells, and lead to type III EMT changes (especially tumors), where the epithelial-mesenchymal transition is closely related to the occurrence and development of gastric cancer (60, 61). Furthermore, fibroblasts exhibit complex immunomodulatory roles in other tumors. In breast cancer, the Yes-associated protein (YAP) pathway promotes fibroblast-induced ECM hardening, and the hardened ECM activates fibroblasts through YAP again, promoting breast cancer progression and immune tolerance (62). Moreover, various cytokines secreted by fibroblasts have complex regulatory effects on T cells, macrophages, and mast cells. Tumor therapy strategies targeting fibroblasts have also been tested in pancreatic cancer, breast cancer, and other tumors (63), Nevertheless, research in this area on GC remains insufficient **(** **Figure 2C** **)**.

### Endothelial cells

Angiogenesis provides nutrition and oxygen to the tumor microenvironment and promotes tumor growth. Endothelial cells play an important role in this process (64). Antiangiogenic vascular endothelial growth factor receptor (VGFR) monoclonal antibody and tyrosine kinase inhibitor (TKI) are also one of the treatment schemes for advanced GC. Single cell sequencing revealed the specificity of tumor endothelial cells (TEC) in TME in phenotype and metabolism. Some TECs have the potential to transform into mesenchymal cells in gastric cancer, and these endothelial cells play an important role in angiogenesis (65). Subsequent studies also showed that TEC participated in the formation of tumor immune tolerance under hypoxia (66). TEC can interact with CAF through VEGFA in GC (67).In other tumors, TEC can up regulate the immune checkpoint molecules of T cells and inhibit the activation of T cells (68). TEC expressing FasL can reduce the number of CD8^+^ T cells and increase the number of Treg (69).

### Mesenchymal stem cells

As a type of pluripotent stem cells, mesenchymal stem cells (MSC) can differentiate into fibroblasts in tumors, and further exhibit a tumor suppressor function in some tumors, which is in contrast with the heterogeneity of MSCs and induced differentiation directions in different tumors (70). The heterogeneity of MSC in the tumor microenvironment of GC must be further clarified **(** **Figure 2C** **)**.

## Endocrine signaling in GC TME

Gastric has endocrine function and can secrete gastrin, cholecystokinin (CCK), secretin and other substances. This is a special characteristic of GC distinct from the other solid tumor, such as lung caner, liver cancer and so on. Which may determine its unique TME for the GC progress. The current research has confirmed that the high expression of gastrin precursor, gastrin, and gastrin downstream receptor CCK2R is an important factor in the occurrence and progression of GC (71). Targeting the gastrin peptide (polyclonal antibody stimulator-PAS) can increase CD8^+^ T lymphocytes and reduce the number of M2 macrophages in GC (72). Gastric endocrine system can promote gastric cancer, but its role in tumor microenvironment and regulation of immune tolerance of gastric cancer still need further research in the future.

## Metabolic heterogeneity GC TME

In gastric cancer TME, the metabolic patterns of various cell components are different. The competition of metabolic substances leads to metabolic reprogramming, thus affecting the function of various cell components in TME, which is one of the reasons for immune tolerance (73).

### Glycolysis

TME lacks nutrients, and glucose metabolism is necessary for cell survival. The glucose uptake capacity of gastric cancer cells is significantly higher than other cells in TME. This glucose deficiency will induce other cells in TME to undergo metabolic reprogramming and then lead to their redifferentiation (73). PTEN-induced kinase 1 (PINK1) deficiency in GC causes M2 polarization of TAMs, which is mainly related to the enhanced glycolysis level caused by PINK1 deletion (74). GC cells overexpressing YAP1 can promote M2 polarization by secreting IL-13, which activates the glucose transporter 3 (GLUT3) dependent glycolytic metabolic reprogramming of TAMs (75, 76). While lactate can further promote the M2 polarization of TAM, inhibition of monocarboxylate transporters (MCT) or HIF-1α can significantly reverse this effect (77), hypoxia-induced elevated glycolysis levels can also induce a decrease in M1 macrophages in GC (78), which suggests an important role of glucose metabolism reprogramming in the tumor microenvironment. Polymorphonucler myeloid-derived suppressor cells (PMN-MDSCs) accumulate in GC cells and inhibit CD8^+^ T cell glycolysis through the S100A8/A9-TLR4/AKT/mechanistic target of rapamycin (mTOR) axis, leading to CD8^+^ T cell exhaustion, and making GC susceptible to PD-1 therapy immune tolerance (79). In GC, the CD155T/TIGIT signaling pathway can inhibit the uptake of glucose by CD8^+^ T cells, thereby inhibiting its function (80). MSC can inhibit the glucose uptake and lactate production of NK cells by upregulating FBP1, thereby weakening their glycolytic metabolism and inhibiting the degranulation ability, perforin production, and cytotoxicity of NK cells (81). In GC, inhibition of Glycogen synthase kinase-3beta (GSK-3β) can increase the infiltration of CD8^+^ T memory stem cells (Tscm) in GC tissue, and promote their differentiation potential and anti-GC ability (82).

High level glycolysis in TME will lead to lactic acid accumulation, induce TME acidification, and inhibit the function of CD8^+^T cells (83). Increase lactic level not only directly limits the cytolytic function of NK cells, but also indirectly inhibits NK cells by increasing the number of MDSC (84). In addition, high level lactic can also induce M2 polarization of macrophages and enhance Treg function (85).

### Lipid

Studies showed that lipid accumulation exists in TAM, and this accumulation of lipids can induce the M2 polarization of TAM, upregulate PD-L1, block the anti-tumor T cell response, and exert an immunosuppressive effect (86). About 30% of tumor-infiltrating lymphocytes (TILs) in the tumor microenvironment of gastric adenocarcinoma are CD69^+^CD103^+^ tissue-resident memory T cells (TRMs) cells. Fatty acid oxidation is necessary for the survival of Trm cells, but the uptake of fatty acids by Trm cells is far less than that of GC cells. The PD- L1 blocker can upregulate the expression of Fabp4/5 in gastric cancer Trm, increase its uptake of fatty acids, and thus enhance its anti-tumor activity (87). CD8^+^CD103^+^TRMs have stronger anti-tumor activity, but exhibit reduced infiltration in gastric cancer, and their cytolytic capacity can be restored by PD-1 blockade and 4-1BB co-stimulation *in vitro* (88).

### Amino acid

Glutamine is necessary for tumor cells, and its metabolites can inhibit the proliferation of T cells and the secretion of cytokines (89). The IDO/Kyn pathway is a classic immunosuppressive pathway. Kyn derived from GC cells can increase the level of Treg infiltration and induce Treg cells to secrete IL-10, which further activates the STAT3/BCL2 pathway to induce GC resistance to chemotherapy (90). Adenosine is a key immunosuppressive metabolite in the tumor microenvironment (91), and Treg cells isolated from peripheral blood of GC patients have the ability to promote adenosine production, which in turn inhibits the activity of CD8^+^ T cells through the A2aR pathway (92).

## Cytokines in GC TME

Cytokines are regulators of innate and adaptive immunity, and play an important role in the formation of tumor immune tolerance. GC cells can secrete IL-17A to promote the transformation of normal fibroblasts (NF) into tumor-associated fibroblasts (CAF). MSC in GC can upregulate the ratio of Tregs and increase the expression of PD-L1 by secreting IL-15, promoting the EMT process and immune tolerance in GC (93). Fibroblasts promote the proliferation of GC cells by secreting IL-8, forming a positive feedback to promote the malignant phenotype of GC access (94). Hepatocyte growth factor (HGF) secreted by CAF can promote angiogenesis of GC through PI3K/AKT and ERK1/2 pathways (95). Elevated serum interleukin 8 (sIL-8) levels are closely related to poor prognosis and lymph node metastasis in GC, sIL- 8 can promote GC metastasis by increasing the PD-1 expression of CD8^+^ T (96). GC-derived TGF-β1 promotes PD-1^-^ independent CD8^+^ T cell dysfunction, and the restoration of CD8^+^ T cell function by combined blockade of PD-1 and TGF-β1 may benefit future GC immunotherapy (97). Cytokines and ECM reorganization in the tumor microenvironment are key factors in the transformation of macrophages from M1 to M2 (98). Studies have shown that TAM can inhibit the function of natural killer (NK) cells in the tumor microenvironment by secreting TGF-β1 (99). M2 macrophage secreted CHI3L1 can play the same role by binding to IL-13Rα2 (100). TAM can activate the NF-κB and STAT3 signaling pathways of GC cells by secreting TNF-α and IL-6, upregulate the expression of PD-L1, and promote the immune escape and proliferation of GC cells (101). The overexpression of secreted acidic cysteine-rich protein (SPARC) in M2 macrophages can inhibit its tumor-promoting effect (102). Studies have also shown that CXCL8 secreted by M2 macrophages can upregulate their own PD-L1 levels (103). IL-4-stimulated EGFR transactivation helps suppress M2 polarization in macrophages, and TAMs in patients with advanced GC have low epidermal growth factor receptor (EGFR) expression, which may be related to the resistance to EGFR monoclonal antibody therapy (104).Tumor-associated neutrophils activate AKT and p38 pathways in MSCs by secreting inflammatory molecules, such as IL-17, IL-23, and TNF-α, inducing their transformation into fibroblasts, and promoting the development of gastric cancer (105). IL-17a produced by TAN can also promote the EMT process of GC through the JAK2/STAT3 pathway (106).

## Noncoding RNAs and exosomes in GC TME

Non coding RNA is a kind of RNA without coding function, including miRNA, lncRNA and circle RNA. Non coding RNA can regulate gene expression and protein function, and form a complex regulatory network. In addition, non coding RNA can also affect other cells in the tumor microenvironment through exosomes, which plays an important role in the formation of tumor immunity. The interaction between Linc00665 and BACH1 leads to the activation and binding of BACH1 to the Wnt1 promoter, promoting M2 polarization of TAMs and GC progression (107). LncRNA CRNDE (108), miR-588 (109), miR-21 (110) in M2-polarized TAM-derived exosomes can enhance the resistance of GC cells to cisplatin, while miR-223 enhances GC cell resistance to doxorubicin (111), Meanwhile, miR-487a, miR-130b-3p can promote the progression of GC (112, 113). M2 macrophage-derived miR-15b-5p can promote GC metastasis through the BRMS1/DAPK1 axis (114). M1-polarized TAM-derived exosomes can down-regulate the expression of PD-L1 in gastric cancer cells through miR-16-5p and activate T cell immunity (115). Linc00152 inhibits CD8 ^+^ T cell infiltration in GC by binding to enhancer of zeste homolog 2 (EZH2) and regulating the CXCL9, 10/CXCR3 axis (116).

Gastric cancer-derived exosomes can induce the PD-1^+^ phenotype in TAMs, most of which will differentiate into M2 macrophages. Furthermore, these exosomes can inhibit the proliferation of CD8^+^ T cells in the microenvironment promotes the secretion of IFN-γ, which in turn promotes the progression of GC (117). Docking protein-1 (DOK1) downregulates the expression of PD-L1 in TAM (118). Another study showed that gastric cancer-derived exosomes could promote the M2 polarization of TAMs through the lncRNA HCG18-miR-875-3p-Kruppel-like factor 4 (KLF4) pathway (119). The overexpression of lncRNA ANCR promotes GC cell invasion and metastasis by inhibiting the polarization of macrophages toward M1 by downregulating Foxo1 (120). In GC, the low expression of miR-128-3p is closely related to the poor prognosis of patients. The direct target of miR-128-3p is IL-16, which can reduce the infiltration of CD4^+^ CD25^+^ Foxp3^+^ Tregs in GC tissue by inhibiting the expression of IL-16 (121). MiR-105-5p is expressed in GC at low levels, and overexpression of miR-105-5p can directly inhibit the expression of PD-L1 and activate CD8^+^ T cells (122). γδ T cells are a class of T cells recently discovered to have important functions in tumor immune tolerance, Vγ9Vδ2 T cells are the main subset of γδ T, and gastric cancer-derived exosome miR-135b-5p can damage anti-tumor function of Vγ9Vδ2 T cells by targeting SP1 (123). MiR-451 in gastric cancer-derived exosomes can transfer to T cells and activate the mTOR pathway, inducing their differentiation into Th-17 cells (124). Gastric cancer-derived exosomal PD-L1 is upregulated in advanced GC patients treated with 5-FU, leading to systemic immune tolerance (125). Mir-1290 in gastric cancer-derived exosomes can inhibit T cell proliferation through the grainyhead-like 2 (Grhl2)/zinc finger E-box binding homeobox 1 (ZEB1)/PD-L1 axis and participate in immune tolerance (126). PD-L1 in exosomes has an immunosuppressive effect in tumors, and histone lysine-specific demethylase 1 (LSD1) can upregulate the level of PD-L1 in gastric cancer-derived exosomes and induce T cell immune resistance (127). GC cell-derived exosomes induce neutrophil autophagy and tumor-promoting activation through the high mobility group box-1 (HMGB1)/TLR4/NF-κB signaling pathway, ultimately promoting the proliferation and migration of GC cells (128). Gastric cancer-derived exosomes can upregulate PD-L1 expression in neutrophils by transporting HMGB1, thereby inhibiting T cell function (129).

## Immunotolerance targeting therapies in GC

Immune cells and non-immune cell components in the microenvironment induce tumor immune tolerance through a variety of mechanisms, which plays an important role in the occurrence and development of GC. Therefore, targeted intervention in the key links of immune tolerance in the microenvironment of GC is expected to become an effective strategy for its treatment. To date, this treatment mainly includes CAR modified cell therapy, herbal medicines, monomer drugs, oncolytic viruses, and other biological agents (**Table 1**).

**Table 1:** Therapeutic strategies targeting tumor immune tolerance in GC microenvironment.

Drugs	Name	Target	Mechanisam	Reference
**CAR-cell**	MSLN-CAR-T	GC	Specifically kill MSLN-positive cells and release cytokines	(130)
**CAR-cell**	cMet-PD1/CD28 CAR-T	GC	Increase the infiltration of central memory T cells	(131)
**CAR-cell**	ICAM-1-CAR-T	GC	Specifically kill ICAM-1-positive cells	(132)
**CAR-cell**	CD133-CAR-T	GC	Target cisplatin-resistant gastric cancer stem cells	(133)
**CAR-cell**	CDH17-CAR-T	GC	Kill tumor cells in a CDH17-dependent manner and do not attack normal epithelial cells	(134)
**CAR-cell**	CLDN18.2-CAR-T	GC	Specifically kill CLDN18.2-positive cells, Phase I clinical trial	(135, 136)
**CAR-cell**	Trop2/PD-L1-CAR-T	GC	Specifically kill Trop2 and PD-L1positive cells	(137)
**CAR-cell**	NK expanded in vitro	GC	Combine with trastuzumab or cetuximab had a certain therapeutic effect on gastric cancer	(138)
**CAR-cell**	PD1-NKG2D-CAR-NK	GC	Enhance whole blood IFN-γ production and reduced peripheral Tregs, Phase I clinical trial	(139)
**CAR-cell**	MSLN-CAR-NK	GC	Specifically kill MSLN-positive cells and enhance NK cell infiltration	(140)
**Monomer drug**	Tranilast	Fibroblasts	Increase the infiltration of CD8+ T cells, and reduce the infiltration of M2 macrophages and mast cells, and reduce proliferation of fibroblasts	(141)
**Monomer drug**	Futibatinib	Fibroblasts	FGFR1-4 inhibitor, Antitumor effect	(142)
**Monomer drug**	Metformin	Fibroblasts	Promote the secretion of Calml3, Antitumor effect	(143)
**Monomer drug**	PPI	Fibroblasts	Inhibit the exosome secretion function of fibroblasts	(144)
**Monomer drug**	Itraconazole	Fibroblasts	Alleviate the resistance of gastric cancer cells to bevacizumab	(145)
**Monomer drug**	IPI549	Macrophages	PI3K-γ inhibitor, restores macrophage function and promotes antitumor T cell responses	(86)
**Monomer drug**	MENK	Macrophages	Promote the M1 polarization, blocking the PI3K/AKT/mTOR signaling pathway	(146)
**Monomer drug**	Pam3Csk4	T cell	TLR2 agonist, active CD8+ T cells	(18)
**Monomer drug**	DAC	T cell	Block DNA methylation in activated PD1+CD8+ TILs	(147)
**Monomer drug**	CCL28 inhibitor	T cell	Inhibit Treg cell infiltration	(148)
**Herbal medical**	Berberine	Macrophages	enhance the phagocytosis of macrophages and therapeutic effects of CD47 antibody and rituximab	(149)
**Herbal medical**	Paeoniflorin	Fibroblasts	Inhibit the secretion of IL-6, Antitumor effect	(149)
**Herbal medical**	Astragaloside IV	Fibroblasts	Inhibit the pathological functions of CAFs	(150)
**Herbal medical**	Triptonide	Fibroblasts	Abolish the ability of GCAFs to induce epithelial-mesenchymal transition	(151)
**Herbal medical**	Sophoridine	Macrophages	Inhibit M2 polarization,increase CD8+ T proliferation and cytotoxic function	(152)
**Herbal medical**	Oleanolic acid	T cell	Promote the balance of Treg/Th17 cells	(153, 154)
**Biological agents**	Oncolytic virus carrying relaxin relaxin	ECM	Degrade ECM components, increase accumulation of cytotoxic T cells and trastuzumab and PD-1 mAbs	(155)
**Biological agents**	Fiber-modified hexon chimeric recombinant oncolytic adenovirus	Fibroblasts	Kill gastric CAFs	(156)
**Biological agents**	Oncolytic herpes simplex virus type 1 virus G47Δ	Macrophages, NK	Decrease M2 macrophages,increase M1 macrophages and NK	(157)
**Biological agents**	PR-Gel	Macrophages, T cell	Increase CD8+ T-cell and M1 infiltration	(158)
**Biological agents**	CD137 antibody	T cell	Enhance CD8+ T cell	(159)
**Biological agents**	iRGD-anti-CD3	T cell	Promote T cell infiltration	(160, 161)
**Biological agents**	sPH20-IgG2	T cell	Enhance the cytotoxicity of MSLN CAR-T	(162, 163)
**Biological agents**	m3s193 BsAb	T cell	Enhance activity in T cell recruiting, activation, proliferation, cytokine release, and cytotoxicity	(164)
**Biological agents**	Hydroxypropyl cellulose photocrosslinked hydrogel incorporating IFN-α2b	T cell	Induce activated T cells into tumor tissue	(165)
**Biological agents**	αPD1-PEG-PCL	T cell	Target PD1+CD8+ TIL	(166)
**Biological agents**	DC cell vaccine loaded with MG-7 antigen	Dendritic cell	Activate specific cytotoxic T lymphocytes	(167)
**Biological agents**	Polylactic-co-glycolic acid nanoparticles encapsulated DC cells and gastric cancer cell soluble lysate	Dendritic cell	Enhance the differentiation of T cells to Th1, enhance the effect of DC vaccine	(168)
**Biological agents**	Dendritic cells modified by SLC	Dendritic cell	Promote DC maturation, enhance the ability of DCs to T cell chemotaxis and T cell stimulation	(169)
**Biological agents**	Heat shock protein -glycoprotein gp96	Dendritic cell, NK	Enhance the antigen-presenting ability of DC	(170)
**Biological agents**	Fusion protein dsNKG2D-IL-15	NK	Recruit and activate NK	(171)
**Biological agents**	Gastrin Vaccine	Gastrin	Increase CD8+ T lymphocytes and reduce the number of M2 macrophages	(72)

### CAR-cell

In recent years, CAR-cell, including CAR-T and CAR-NK had been developed for treating GC. Mesothelin (MSLN)-CAR-T cells can effectively inhibit the growth of GC cells in the Patient-derived tumor xenograft (PDX) model (130). Mesenchymal-epithelial transition factor (cMet)-PD1/CD28 CAR-T is a second-generation CAR. The researchers constructed a PD1/CD28 chimeric switch receptor by fusing the extracellular domain of PD-1 with the transmembrane and intracellular domains of CD28. Converting the inhibitory signal of PD-1 into the activation signal of CD28 can effectively inhibit the growth of GC *in vitro* and *in vivo*, and increase the infiltration of central memory T cells, prolong the long-term anti-tumor effect, and reducing the secretion of inflammatory factor IL-6 (131). The intercellular adhesion molecule 1 (ICAM-1) is expressed in nearly 50% of GC patients. In mouse models, CAR-T cells targeting ICAM-1 can target both primary and metastatic gastric cancers, exhibiting a good therapeutic effect (132). In a mouse model, anti-CD133 chimeric antigen receptor T (CAR-T) can selectively target cisplatin-resistant GC stem cells, and the combined use of cisplatin can improve the therapeutic effect (133). Nanobody VHH1-driven CAR-T (CDH17CART) targeting CDH17 can effectively treat gastrointestinal tumors without affecting normal epithelial cells in mouse experiments (134). Claudin18.2 (CLDN18.2) is a gastric-specific membrane protein, and CLDN18.2-specific CAR-T cells can effectively partially or completely eliminate GC in the PDX model. To date, this treatment passed phase I clinical trials (135, 136). Bispecific trophoblast cell surface antigen 2 (Trop2)/PD-L1-specific third-generation CAR-T cells were developed through lentiviral infection, which can effectively kill GC cells *in vitro* (137). A phase I clinical trial showed that *in vitro* expanded NK cells combined with trastuzumab or cetuximab had a certain therapeutic effect on GC, which was well tolerated by patients (138). A dual-targeting chimeric receptor (DTCR) PD1-DAP10/NKG2D increases the expression of PD1 and NKG2D on the surface of NK92 cells by viral transfection, and has comparable anti-tumor properties in a mouse tumor-bearing model constructed with SGC-7901 activity (139). MSLN-CAR NK cells constructed based on NK-92 cells can effectively kill MSLN-positive GC cells *in vitro* and inhibit tumor growth in the PDX model, with a large number of NK cells infiltrating the tumor (140). CAR cells constructed from various immune cells have shown a certain curative effect in animal models of GC. However, whether these treatments are effective for GC patients, and whether the selection of patients is targeted for their application, still needs extensive research.

### Monomer drug

Studies have shown that the antiallergic drug Tranilast can inhibit the secretory function of fibroblasts in peritoneal metastatic GC tissue, effectively improve the tumor microenvironment, increase the infiltration of CD8^+^ T cells, as well as reduce the infiltration of M2 macrophages and mast cells. This leads to reduced proliferation and fibrosis of GC cells (141). Futibatinib is a novel FGFR1-4 inhibitor that exhibits broad-spectrum antitumor effects in various tumors, including gastric cancer (142). Metformin can promote the secretion of calmodulin-like protein 3 (Calml3) from CAF cells and inhibit the progression of GC (143). In patients with advanced GC, the application of large doses of PPI can inhibit the exosome secretion function of fibroblasts, improve the tumor microenvironment, and inhibit the malignant degree of GC (144). Nevertheless, the effect of PPI on gastric cancer remains controversial. Itraconazole can inhibit the activity of endothelial cells and fibroblasts in GC and alleviate the resistance of GC cells to bevacizumab (145). A selective inhibitor of PI3K-γ isoenzyme, IPI549, restores macrophage function and promotes anti-tumor T cell responses (86). Experiments *in vivo* show that methionine enkephalin (MENK) can promote M1 polarization of macrophages and upregulate the expression of opioid receptor (OGFr) by blocking the PI3K/AKT/mTOR signaling pathway, which inhibits GC cells (146). In CD8^+^ cells isolated from peripheral blood of tumor patients, the TLR2 agonist Pam3Csk4 enhanced the cytolytic activation of peripheral and tumor-infiltrating CD8^+^ T cells from GCs (18). *De novo* DNA methylation is acquired by PD1^+^CD8^+^ tumor-infiltrating T cells (TILs), which results in graded downregulation of cytokines, such as interferon-γ (IFN-γ), while 5-Aza-2’-deoxycytidine (DAC) *de novo* blocks DNA methylation in activated PD1^+^CD8^+^ TILs (147). The CCL28 blockade inhibits Treg cell infiltration and tumor progression in the mouse model (148). The single drugs mentioned above affect tumor immune tolerance through different mechanisms, thus playing a certain role in the treatment of GC. However, the targeting problem of monomeric drugs *in vivo* may be dangerous, diminishing the therapeutic effect and even causing serious side effects. Improving the targeting precision of their actions, such as by combining them with monoclonal antibodies, may solve this problem.

### Herbal medicine

Herbs are widely used as tumor immune regulators and chemotherapeutic sensitizers. Although the therapeutic effect of herbs and their natural compounds is not as significant as that of classical drugs, their advantages of low toxicity and low side effects endow them with potential in tumor treatment (172, 173). In addition, some herbal medicines play an important role in the metabolic regulation of gastrointestinal tumors (174).

Berberine has complex functions in gastrointestinal tumors, including including autophagy, immunity, inflammation, modification of the gut microbiota and miRNA. Berberine is an inhibitor of CD47, which can enhance the phagocytosis of macrophages, and enhance the therapeutic effects of CD47 antibody and rituximab (149). Paeoniflorin improves the immune microenvironment of GC and inhibits the invasion and metastatic ability of GC by inhibiting the secretion of IL-6 in fibroblasts in GC tissue (175). Astragaloside IV and Triptonide can inhibit the cancer-promoting function of fibroblasts in GC (150, 151). Sophoridine inhibits M2-TAM polarization *via* the TLR4/IRF3 axis, increases CD8^+^ T proliferation and cytotoxic function, and downregulates the expression of CD8^+^ T cell exhaustion markers PD-1, Tim-3, and Lag-3 (152). Oleanolic acid can promote the balance of Treg/Th17 cells in GC by targeting IL-6 through miR-98-5p, and is a potential drug for the treatment of GC (153, 154).

### Other biological agents

Several researchers constructed an oncolytic virus carrying relaxin (RLX), which can degrade ECM components in tumors (155). Results showed that in *in vivo* experiments, the oncolytic virus carrying RLX could effectively degrade the ECM of gastric cancer and increase the activated ECM. The accumulation of cytotoxic T cells and trastuzumab and PD-1 mAbs in gastric cancer tissues yielded significant anti-tumor effects (155). A fiber-modified hexon chimeric recombinant oncolytic adenovirus targeting CAFs can relatively specifically kill gastric CAFs and inhibit GC cell growth *in vivo* (156). G47Δ, a third-generation oncolytic herpes simplex virus type 1 virus, has passed phase II clinical trials in glioma, and has demonstrated significant anticancer effects in orthotopic tumor models and peritoneal dissemination models of GC. M2 macrophages were decreased, while M1 macrophages and NK cells were increased (157). A research group developed an injectable shear-thinning hydrogel, co-loaded with polyphyllin II (PP2) and resiquimod (R848) (PR-Gel for short), which induces TAM cell M2 in a mouse model of GC. Enhanced repolarization and CD8^+^ T cell infiltration to M1 exhibited favorable tumor suppressive effects (158). In *in vitro* experiments, the CD137 antibody can effectively induce apoptosis in primary GC cells by enhancing CD8^+^ T cells *via* activation of NF-κB signaling (159). A novel tumor-penetrating peptide, iRGD-anti-CD3, can immobilize iRGD on the surface of T cells through CD3 binding, promote T cell infiltration, and increase T cell activation and cytotoxicity to target cancer cells in 3D culture models and *in vivo* experiments (160, 161). Replacing the PH20 signal peptide with the tPA signal peptide and linking the IgG2 Fc fragment to construct human hyaluronidase PH20 (referred to as sPH20-IgG2) can enhance the cytotoxicity of MSLN CAR-T against GC in a mouse model (162, 163). Targeting Lewis Y and CD3 (m3s193 BsAb), a formatted novel T cell-binding bispecific antibody with IgG-[L]-scfv exhibited promising anti-GC activity in a mouse huPBMCs/GC co-transplantation model (164). Hydroxypropyl cellulose photocrosslinked hydrogel incorporating IFN-α2b can ensure the activity of IFN-α2b, stably release IFN-α2b to stimulate T cells over a long time, and combined with low-dose radiation of 5 Gy can induce activated T cells into tumor tissue, increasing the immunotherapy effect (165). The conjugation of αPD1 (i.e., nivolumab) to poly(ethylene glycol) (PEG) and poly(ϵ-caprolactone) (PCL) copolymers with PEG as linker (αPD1-PEG-PCL) by double emulsion solvent evaporation, encapsulating DAC in αPD1-PEG-PCL, this drug can better target PD1^+^CD8^+^ TIL to inhibit and kill GC cells (166). Anti-TGF-β/PD-L1 bispecific antibody YM101 is superior to anti-TGF-β and anti-PD-L1 monotherapies, increasing the numbers of tumor infiltrating lymphocytes and dendritic cells, elevating the ratio of M1/M2, and enhancing cytokine production in T cells (176). As an agonist of STING, bivalent manganese (Mn2+) can cooperate with YM101 to produce more lasting anti-tumor effect and enhance the presentation of tumor antigen (177). M7824 (MSB0011359C) is a bifunctional fusion protein composed of a monoclonal antibody against PD-L1 fused to the extracellular domain of TGF-β receptor II, the dual anti-immunosuppressive function of M7824 resulted in activation of both the innate and adaptive immune systems, which contributed to M7824’s antitumor activity relative to monotherapies (178). The above three bioagents had been confirmed that they could suppress multiple tumor cell lines, including colon cancer, lung cancer, breast cancer. Although it is not reported that their roles in GC, it provides potential therapy to GC in future. The DC cell vaccine loaded with MG-7 antigen (MG-7Ag) significantly activates specific cytotoxic T lymphocytes in the GC PDX model (167). Polylactic-co-glycolic acid nanoparticles (NPs) are encapsulated by DC cells, and the GC cell soluble lysate can enhance the differentiation of T cells to Th1 in tumors and enhance the effect of the DC vaccine (168). Dendritic cells modified by the SLC gene can promote DC maturation, enhance the ability of DCs to T cell chemotaxis and T cell stimulation, and induce specific anti-GC cellular immunity (169). The heat shock protein (HSP)-glycoprotein (gp) 96 can enhance the antigen-presenting ability of DC cells and the activity of NK cells *in vitro* (170). The fusion protein dsNKG2D-IL-15 can recruit and activate NK cells and inhibit the growth of GC in a nude mouse model (171).

## Conclusion

In fact, there is no lack of immune cells in tumors, including immune cells with tumor suppressor functions. However, the exhaustion and functional inhibition of these cells are fundamental reasons for tumor immune tolerance and immune escape. In-depth research is particularly important for tumors such as GC, which have a large patient base and few treatment options. However, current research on tumor immunity of GC is significantly less than that of lung, colon, and other cancers, and numerous classical mechanisms of action have not been confirmed in GC. This may be related to the unique anatomical characteristics of gastric tissue. As an important part of the digestive system, the stomach is in close contact with various foods ingested from the outside world, thus maintaining its immune tolerance to food. At the same time, due to the long-term high gastric acid environment, Whether it also constitutes a unique immune microenvironment in the stomach may be inseparable from the occurrence and development of GC. The immune escape of gastric cancer is closely related to its tumor microenvironment, especially considering the changes in metabolic patterns and metabolites of each cell component in it, as well as the role of the secretion of each cell component. The dynamic changes in the tumor microenvironment in the tumor-promoting direction during tumor progression are of great significance. Reversing the reprogramming of the tumor microenvironment to counter the tumor-promoting direction can effectively solve the immunosuppression of tumors. Numerous traditional medicines have also been found to exert anti-tumor effects, which suggests that we must pay attention to the possibility of traditional medicines and traditional Chinese medicines as immunomodulators and sensitizers in tumor treatment. Considering the currently popular CAR-T, the main problems involve making CAR-T effectively infiltrate solid tumor tissue, making CAR-T play a lasting role in the immunosuppressive tumor microenvironment, and finding effective chimeric targets in as many tumor cells as possible, newer modifications and gene targets remain to be developed. NK cells and neutrophils as emerging therapeutic directions in GC must also be further studied.

With the development of omics technology, the heterogeneity of GC and various cellular components in GC TME are well known to us. Their differences are the only way to find solutions to the immune tolerance of GC and achieve precision medicine. Future research must explore the relationship and differences between GC and TME from a holistic perspective, find targets for the overall tumor microenvironment, and determine novel directions for solving GC immune tolerance.

## Author contributions

CFL and WY conceptualized the study. YDL drafted the manuscript. YPL and CL conducted the literature review. All authors contributed to the article and approved the submitted version.

## Funding

This review was supported by grants from the Natural Science Foundation of China (No. 81872323, No. 82073299), Finance Department of Jilin Province (2021SCZ12, 2019SCZ013) and project of Health Commission of Jilin Provincial (No. 2021JL035).

## Conflict of interest

The authors declare that the research was conducted in the absence of any commercial or financial relationships that could be construed as a potential conflict of interest.

## Publisher’s note

All claims expressed in this article are solely those of the authors and do not necessarily represent those of their affiliated organizations, or those of the publisher, the editors and the reviewers. Any product that may be evaluated in this article, or claim that may be made by its manufacturer, is not guaranteed or endorsed by the publisher.
